# Case Report: Proton beam therapy – friend or foe for patients with *IDH*-mutated WHO grade 2 and 3 gliomas?

**DOI:** 10.3389/fonc.2026.1803943

**Published:** 2026-05-08

**Authors:** Liv Cathrine Heggebø, Lars Fredrik Fjæra, Frank Leonel Bello Garrote, Ida Maria Henriksen Borgen, Hanne Blakstad, Taran Paulsen Hellebust, Cathrine Saxhaug, Kjetil Knutstad, Hillevi Rylander, Thomas Henry, Magnus Gustafsson, Katja Werlenius, Malin Blomstrand, Petter Brandal

**Affiliations:** 1 Department of Oncology, Oslo University Hospital, Oslo, Norway; 2 Institute of Clinical Medicine, University of Oslo, Oslo, Norway; 3 Department of Medical Physics, Oslo University Hospital, Oslo, Norway; 4 Department of Physical Medicine and Rehabilitation, Oslo University Hospital, Oslo, Norway; 5 Department of Radiology, Oslo University Hospital, Oslo, Norway; 6 The Skandion Clinic, Uppsala, Sweden; 7 Department of Medical Physics and Biomedical Engineering, Sahlgrenska University Hospital, Gothenburg, Sweden; 8 Department of Medical Radiation Science, Institute of Clinical Sciences, Sahlgrenska Academy, University of Gothenburg, Gothenburg, Sweden; 9 Department of Oncology, Institute of Clinical Sciences, Sahlgrenska Academy, University of Gothenburg, Gothenburg, Sweden; 10 Department of Oncology, Sahlgrenska University Hospital, Gothenburg, Sweden

**Keywords:** case report, *IDH*-mutated glioma, oligodendroglioma, proton therapy, radionecrosis, RICE

## Abstract

**Background:**

Proton beam therapy is increasingly implemented for lower-grade gliomas worldwide, aiming to reduce radiation exposure to healthy tissue and lower treatment related toxicity. However, robust clinical evidence of the benefit of radiotherapy delivered with protons compared to photons is lacking. Importantly, although proton therapy offers potential benefits compared to photon radiotherapy, it is still ionizing radiation and can be associated with severe complications. Emerging concerns include a possible increase of radiotherapy-induced contrast-enhancing lesions.

**Case presentation:**

A healthy woman in her mid-forties underwent a subtotal resection for an *IDH-*mutated oligodendroglioma CNS WHO grade 2. She was randomized to proton therapy in the PRO-GLIO trial and received a dose of 54 Gray (Gy) relative biological effectiveness (RBE), followed by chemotherapy. Ten months after completion of radiotherapy, she reported rapid visual deterioration ultimately resulting in blindness. She also developed substantial cognitive deficits, pituitary and hypothalamic failure, and a general decline. MRI showed considerable, progressive radionecrosis in large parts of the irradiated brain. Tragically, the patient passed away 22 months after completing proton therapy.

**Conclusion:**

This case highlights that although proton beam therapy is considered safe and potentially encumbered with fewer side effects than photon radiotherapy, serious complications may occur. Careful consideration of timing and execution of adjuvant therapy for lower-grade gliomas is essential. Randomized controlled trials are necessary to disclose if proton beam therapy is beneficial or not in lower-grade gliomas.

## 1 Introduction

Lower-grade isocitrate dehydrogenase (*IDH*)-mutated gliomas are principally incurable diseases of which four subtypes exist; oligodendroglioma CNS WHO grade 2 and 3 and astrocytoma CNS WHO grade 2 and 3 (1). Although incurable, these neoplasms have a slow growth pattern, life expectancy is long and affected patients are relatively young, often diagnosed in their fourth or fifth decade (2–5). Median overall survival varies from 12.5 years for patients with *IDH*-mutated astrocytoma grade 3 (6) to approximately 18 years for individuals with *IDH*-mutated oligodendroglioma grade 2 (7). Anti-neoplastic treatment consists of surgery, radiotherapy, chemotherapy, and *IDH*-inhibitor (2, 3, 6–9). Although the *IDH*-inhibitor vorasidenib has been approved for *IDH*-mutated glioma grade 2 by the U.S. Food and Drug Administration (FDA) and the European Medicines Agency (EMA), it has not yet received national approval in all countries, including Norway and Sweden. Timing of treatment is important to maximize effect, as well as to postpone treatment-related toxicity. Active surveillance following primary surgery is often chosen for grade 2 neoplasms, although some evidence suggests that early anti-neoplastic treatment is beneficial for tumor control and maintaining health-related quality of life (8, 9). Higher grade, older patient age (>40–50 years), residual tumor, and persistent neurological deficits all argue for early anti-neoplastic treatment, however, each patient needs to be evaluated individually (8, 9).

Radiotherapy, although an effective anti-neoplastic treatment, is encumbered with multiple and potentially serious adverse effects (4, 9–12). Proton beam therapy (PBT) with its characteristic Bragg peaks enables better sparing of healthy tissue from radiation exposure compared to its photon counterpart, theoretically reducing the risk for treatment-related toxicity (4, 10, 12). Clinical evidence of benefit, and its magnitude, is, however, lacking (4, 10, 13). Also, PBT is not as available as photon radiotherapy (XRT). For most patients treatment with PBT necessitates time spent away from home, and if the clinical benefit is small or even non-existent this burden results to substantial time toxicity (14). Furthermore, in the case of lower-grade gliomas, some but not all reports have raised concerns about radionecrosis, frequently referred to as radiation-induced contrast-enhancing lesions (RICE) following PBT (15–17).

We present a case of extensive RICE with fatal outcome for a patient following PBT for an *IDH-*mutated grade 2 oligodendroglioma. The patient presented with a focal epileptic seizure and was otherwise in excellent general condition without neurological deficits. A near gross total resection of her left frontal lobe neoplasm was performed. She was offered and accepted inclusion in the ongoing phase 3 PRO-GLIO trial, one of three trials randomizing patients with *IDH-*mutated gliomas grade 2 and 3 to proton or photon radiotherapy [NCT03180502, (18, 19)].

## 2 Case presentation

**Figure 1:**
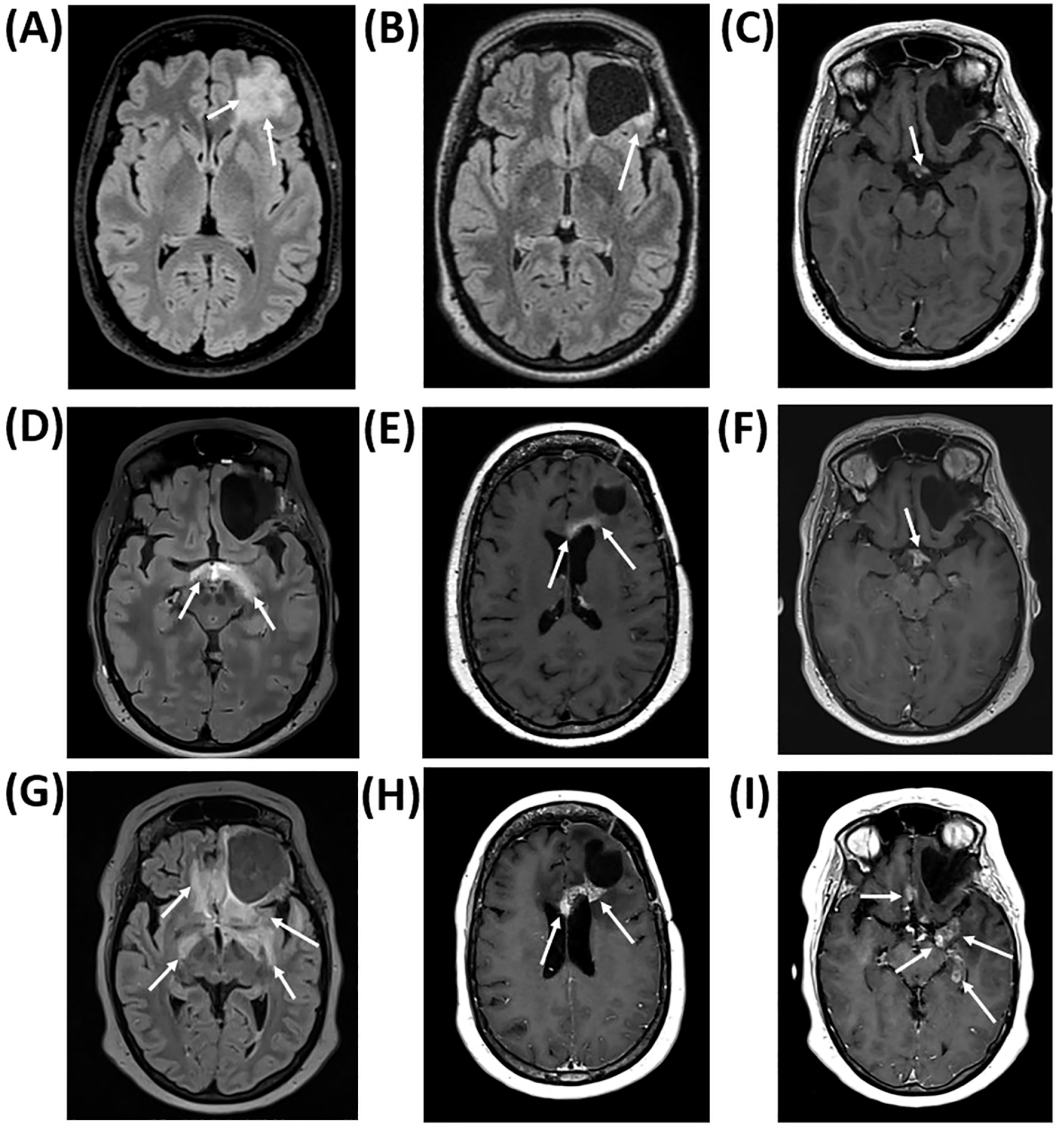
Axial magnetic resonance imaging (MRI) scans. **(A)** displays a left-sided, frontal, diffuse T2-weighted fluid-attenuated inversion recovery (FLAIR) hyperintense and gadolinium non-enhancing lesion in the preoperative MRI scan. **(B)** Postoperative T2-weighted/FLAIR image showing a small residual tumor (10 millimeters) dorsolateral to the resection cavity. **(C)** displays subtle chiasmatic contrast enhancement in a T1-weighted MRI-series at symptom debut 9.5 months following completion of radiotherapy, **(D)** shows FLAIR hyperintensity approximately one month after symptom debut, and **(E, F)** show contrast enhanced T1-weighted image sequences six months from symptom debut, with radiation-induced contrast enhancement in optic pathways and genu corporis callosi. **(G–I)** display T2-weighted/FLAIR hyperintensity changes and increasing contrast enhancement in an MRI scan 12 months from symptom debut and two weeks before the patient passed away, corresponding to 21 months after radiotherapy completion. Arrows are used to draw attention to pathology in all images.

A previously healthy woman in her mid-forties was admitted to hospital following a focal epileptic seizure. Magnetic resonance imaging (MRI) revealed a diffuse lesion suspicious of an *IDH*-mutated glioma in the left frontal lobe, **Figure 1A**. She underwent a subtotal resection, leaving a residual tumor of 10 millimeters, **Figure 1B**. Histopathology confirmed an *IDH-*mutated oligodendroglioma grade 2 with a 1p/19q codeletion. Her general condition was excellent with an Eastern Cooperative Oncology Group (ECOG) status of 0 and a Neurologic Assessment in Neuro-Oncology (NANO) score of 0.

Active surveillance or initiation of further anti-neoplastic therapy were considered. Following discussions in the multidisciplinary team and with the patient, a decision to initiate adjuvant therapy was made. The patient consented to participation in the PRO-GLIO trial and a pre-radiation neuropsychological evaluation revealed only minor cognitive difficulties (variable attention function and lightly reduced psychomotor speed), and otherwise average to high average cognitive functioning. She was randomized to PBT and received 1.8 Gray (Gy) relative biological effect (RBE) × 30, which was well tolerated except for a localized skin rash. Chemotherapy with procarbazine, lomustine, and vincristine (PCV) was initiated four weeks after completion of radiotherapy, and a total of five courses were administered. The patient experienced fatigue common terminology criteria for adverse events (CTCAE) grade 2 and bone marrow suppression grade 2–3 during chemotherapy.

**Figure 2:**
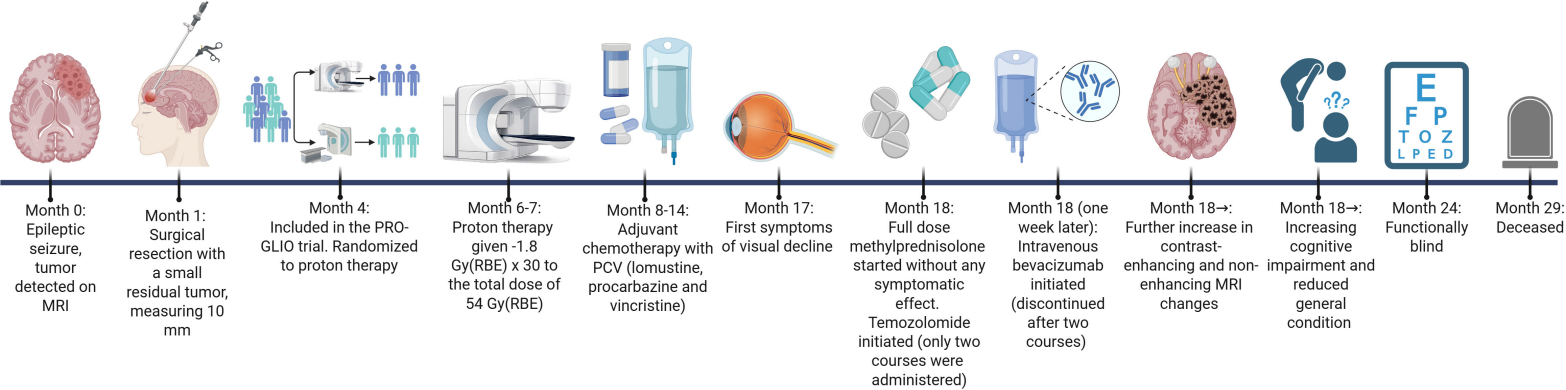
Timeline illustrating the patient’s clinical journey. Month 0 is set at the time of diagnosis. Gy, Gray; mm, millimeter; MRI, magnetic resonance imaging; RBE, relative biological effect.

Ten months following completion of PBT, the patient reported reduced vision. Ophthalmologic examination revealed right-sided homonymous hemianopsia and papilledema in the left eye. MRI was first described as unchanged, however, subtle contrast enhancement was seen retrospectively, **Figure 1C**. One month later, MRI showed radiological changes in optic nerves, chiasm, optic tracts, and hypothalamic region, **Figure 1D**. High-dose methylprednisolone (64 milligrams daily) was initiated without clinical improvement. As vision continued to deteriorate, bevacizumab (7.5 milligrams/kilogram) was initiated one week later but was discontinued after two courses due to malignant hypertension and proteinuria requiring hospitalization. Temozolomide was also attempted as neoplastic progression could not be fully ruled out, however, discontinued after two cycles due to consolidation of the RICE diagnosis using MRI perfusion and diffusion. Amino acid positron emission tomography (PET) is not routinely used at OUS. The next MRI scan showed transient radiological improvement likely related to the administered bevacizumab, but visual deterioration continued and the patient was functionally blind seven months after symptom onset. Subsequent MRI scans revealed progressive RICE, **Figures 1E–I**. The patient exhibited marked cognitive decline, accompanied by loss of functional independence and delirium, leading to need for institutionalization. At this stage, she lacked the capacity to undergo formal neuropsychological assessment. In few months the patient developed general deterioration and pituitary and hypothalamic failure resulting in diabetes insipidus, hypernatremia, and appetite dysregulation. She was treated with desmopressin, levothyroxine, and corticosteroid replacement therapy. Nonetheless, clinical deterioration progressed and the patient passed away 12 months from debut of her visual symptoms. Autopsy revealed extensive radionecrosis involving large regions of the brain, concluded to be the primary cause of death. The patient’s medical journey is outlined in **Figure 2**.

Admission to Department of Neurology for second opinion was performed twice, first two months after onset of visual symptoms and again four months later. Apart from slightly elevated protein levels in the cerebrospinal fluid, likely related to the radionecrosis process, no pathological findings were detected. Genetic testing using a Next-Generation Sequencing (NGS) gene panel for hereditary cancer revealed a mosaic *TP53*-mutation of uncertain clinical significance, but no known radiotherapy vulnerability predisposing syndrome. The patient’s family was offered genetic counselling following the results from the genetic testing.

**Figure 3:**
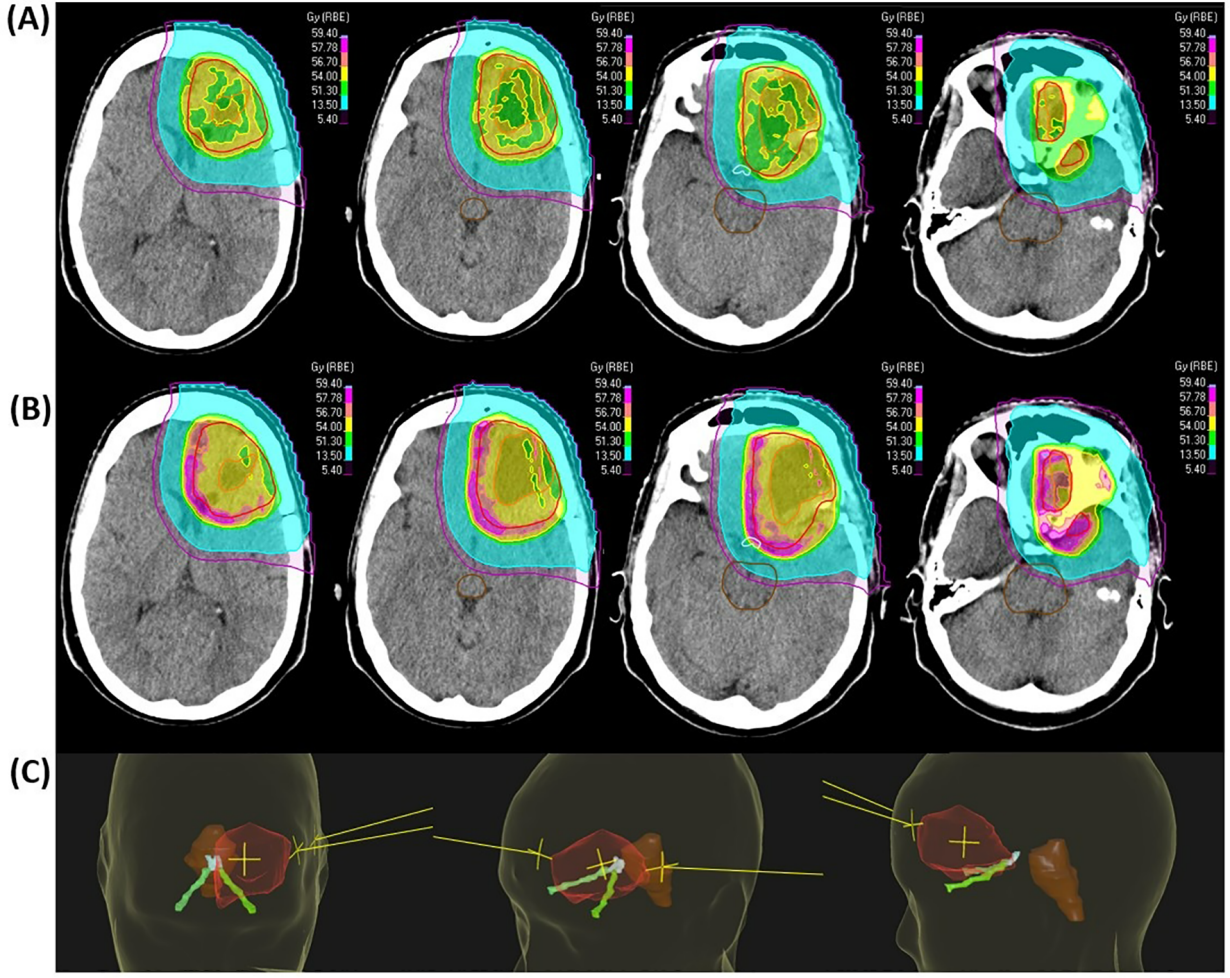
The patient’s proton plan showing isodose levels, radiotherapy target volumes and some OARs. **(A)** displays nominal doses and **(B)** calculated linear energy transfer (LET) weighted doses. GTV includes the resection cavity and the suspected residual tumor (orange). A margin of 10 millimeters was added to GTV to define CTV (red). CTV was modified against natural anatomical barriers. OARs: brainstem (brown), optic nerves (green) and chiasm (blue). The 105% (56.7 Gy(RBE)) isodose volume is shown in pink, 95% (51.3 Gy(RBE)) in yellow, 50% (27.0 Gy(RBE)) in green, 25% (13.5 Gy(RBE)) in turquoise, and 10% (5.4 Gy(RBE)) in purple. **(C)** displays beam angles for the patient’s proton plan in relation to CTV (transparent red), brainstem (brown), and optic apparatus (optic nerves in green and chiasm in blue). On the left side, the first beams eye view is illustrated with a gantry angle of 340° and a couch angle of 90°, delivering 156 monitor units (MU). The middle figure shows the second beams eye view with a gantry angle of 55° and a couch angle of 335°, delivering 148 MU, and the right sided figure displays the third beams eye view with a gantry angle of 107° and a couch angle of 350°, delivering 167 MU. CTV, clinical target volume; GTV, gross tumor volume; Gy, Gray; mm, millimeter; MRI, magnetic resonance imaging; OARs, organs at risk; RBE, relative biological effect.

Based on the unexpected and severe treatment-related toxicity, hospital legal authorities and relevant governmental bodies were notified. The PBT plan underwent extensive scrutinization in the PRO-GLIO study group, national governmental bodies, and also in two independent PBT institutions outside Norway and Sweden. All evaluations concluded that PBT had been administered according to current standards, with appropriate target coverage and OARs sparing, and without dosimetric hot spots. PBT had been delivered with three fields aiming for optimal angulation to achieve a conformal and homogenous dose distribution, **Figure 3C**. Robust evaluation was performed according to the PRO-GLIO protocol and standard clinical practice, using +/- 3 millimeters and a range of +/- 3.5%. Doses to OARs were within constraints defined by the European Particle Therapy Network (EPTN) consensus (20), **Table 1**. The clinical target volume (CTV) was 77.3 cubic centimeters. The CARE Checklist was applied when writing this case report, see **Supplementary Material**.

**Table 1:** Doses to key structures.

Structure (metric)	Dose (Gy(RBE))	EQD2 dose (α/β=2 Gy) (Gy(RBE))	EPTN consensus based EQD2 dose constraints (Gy)
Left hippocampus(D_40%_)	1.8	0.9	≤7.3
Right hippocampus (D_40%_)	0.0	0.0	≤7.3
Optic chiasm (D_0.03cc_)	53.4	50.5	≤55
Left optic nerve (D_0.03cc_)	53.7	50.9	≤55
Right optic nerve (D_0.03cc_)	44.3	38.5	≤55
Body (patient contour), (D_0.03cc_)	56.8	55.2	NA

α/β, alfa/beta; CTV, clinical target volume; D_0.03cc_, maximum dose; D_40%_, dose to 40%; EPTN, European Particle Therapy Network; EQD2, equivalent dose in 2 Gy fractions; Gy, Gray; RBE, relative biological effect. All doses are nominal.

## 3 Discussion

Following PBT, a previously healthy woman with an *IDH*-mutated oligodendroglioma grade 2 experienced extensive RICE. This led to blindness, hypothalamic and pituitary failure, cognitive decline, and ultimately death. The case blatantly highlights that also PBT, although with diminished radiation exposure of normal tissue compared to photon radiotherapy, nonetheless is encumbered with a risk of severe treatment-related toxicity analogous to photon radiotherapy.

Radiotherapy is an effective anti-neoplastic treatment modality in diffuse gliomas (2, 21), and is extensively used in *IDH*-mutated grade 2 gliomas. A major caveat with radiotherapy, however, is the risk of unwanted effects including potentially severe late sequelae (10–12). As patients with *IDH*-mutated grade 2 gliomas are young and with a favorable prognosis, reduction of unwanted late effects is a high priority. PBT has inherent properties enabling better sparing of healthy tissue from radiation exposure when compared to XRT (4, 13, 22). Dosimetrically, it looks evident that PBT is superior to XRT for lower-grade gliomas (10, 18, 22). However, there are no high-quality data supporting this position (10, 13), and several arguments could be made against such a perspective. The diffuse infiltration of glioma cells into seemingly normal brain tissue suggests that PBT might lead to undercoverage of neoplastic cells (18, 23), and there are still uncertainties associated with PBT. Internationally, as well in this case, RBE is typically fixed at 1.1. However, the RBE is not invariant and depends on factors such as the linear energy transfer (LET). LET increases toward the distal end of the Bragg peak, so using a fixed RBE of 1.1 in these areas might underestimate the biological dose. Regions characterized by high LET, and consequently increased biological dose, are associated with an elevated risk of toxicity, including RICE (24, 25). Several reports on unpredictable toxicity in patients with lower-grade gliomas following PBT remind us of this and is of concern (16, 17, 26).

However, the reports on unexpected toxicity are based on retrospective data, and RICE occurs after both PBT and XRT. For lower-grade gliomas, RICE is reported in 12-34% of cases following PBT (15, 25, 27–29) and in 10-28% of cases following XRT (28–31). Most RICE cases are mild and asymptomatic (4), usually appear within the radiotherapy target volume (25, 27, 30), and the periventricular zone (PVZ) seems to be a predilection site (15). For our patient, the first RICE lesion appeared within the optic apparatus and was located outside of both the target volume and the PVZ. Later RICE changes occurred, however, within the PVZ. Most studies do not report higher toxicity from RICE than CTCAE grade 3 (15, 25, 27, 29, 31), however, a few higher grades of RICE have been reported (28). The fatal outcome of our patient is exceptional, and to the best of our knowledge not previously reported following standard dose PBT for an *IDH*-mutated grade 2 glioma. The risk of RICE correlates with radiation dose and irradiated brain volume, and some studies link older age, grade 2 histology, and re-irradiation to higher risk of RICE (4, 27–31). Our patient harbored a 1p/19q codeletion, which in some but not all reports is associated with an increased risk of RICE (28, 30–32).

In our patient, RICE appeared 10 months following radiotherapy and mainly outside the target volume. RICE increased and the patient developed blindness (RION – radiation induced optic neuropathy), cognitive decline and ultimately had a fatal outcome. The PBT plan had OAR doses well below constraints, and independent second and third opinions obtained on the treatment plan identified no flaws. We decided to perform retrospective analyses on LET-weighted dose, using the formula *D_biological_ = D_physical_ × RBE = D_physical_ × (1 + LET_d_ × c)*, where LET_d_ is the dose-averaged LET. The *c* is a scaling factor defined as *c = 0,055 µm/keV* published by McMahon et al. (33). The LET_d_ was calculated in water for primary and secondary protons using Monte Carlo in RayStation^®^ version 2024A SP1. The analyses of our case showed an elevated LET-weighted dose with a maximum (dose to 0.03 cubic centimeters D_0.03cc)_) of 59.1 Gy(RBE) in the area where RICE first appeared, acknowledging the fact that these analyses are based on models and parameters with considerable uncertainties. Nevertheless, this might explain parts of the clinical picture, as the calculated LET-weighted dose is significantly higher than the nominal RBE 1.1 dose. The RICE lesion appeared predominantly outside the target volume, however, with an increased LET-weighted dose in this area. Elevated LET combined with high doses within the locations where RICE occurs are similar to findings from others (17, 25, 34). Genetic analyses and autopsy revealed no explanation for the extensive tissue damage, leaving only speculation about an unknown genetic vulnerability. Although the patient only had a mosaic *TP53* mutation, Li-Fraumeni syndrome harboring germline pathogenic variants in *TP53* might potentially lead to higher radiosensitivity, including an increased risk of radiation-induced malignancies and therefore likely benefiting from PBT because of the dosimetric advantages (35). Our patient was offered combined radiotherapy and chemotherapy which prolonged median overall survival from 7.8 to 13.3 years in patients with *IDH*-mutated grade 2 glioma in a previous study (2). This effect needs to be weighed against unwanted side effects which may be devastating. It was, though, impossible to anticipate that this patient would develop severe RICE. PBT reports indicate similar dose constraints for RION as XRT (36), and we believe it is probable that the patient would have developed RICE also if she had received XRT.

We argue that randomized studies need to be carried out to shed light on the hitherto unquantified possible benefit of proton, or photon, radiotherapy compared to the other modality in patients with lower-grade gliomas. One such study has completed enrollment and two are ongoing [NCT03180502, (18, 19)]. It is of course very important not to endanger patients and protective measures such as dose lowering, LET evaluation, and extra caution to avoid high radiotherapy doses in the PVZ and other critical organs need to be discussed. Further studies are necessary to evaluate biological and clinical uncertainties related to PBT; is the magnitude of a potential benefit enough to avoid time and financial toxicity for patients, caregivers, and society (13, 14).

In conclusion, our patient developed extensive symptomatic RICE and tragically passed away 22 months after completion of PBT. This case underscores that, although hopefully encumbered with fewer and less severe unwanted side effects, PBT is – as is photon radiotherapy - ionizing radiation with a risk of grave complications. Randomized studies should be executed to reveal if and to what extent PBT is beneficial, particularly in lower-grade gliomas. Careful consideration of timing of adjuvant therapy for lower-grade gliomas is also essential. In addition, we advocate that LET and RBE considerations should be incorporated into PBT planning, although existing models remain associated with considerable uncertainty.

## Data Availability

The original contributions presented in the study are included in the article/**Supplementary Material**. Further inquiries can be directed to the corresponding author.

## References

[1] Louis DN Perry A Wesseling P Brat DJ Cree IA Figarella-Branger D . The 2021 WHO classification of tumors of the central nervous system: a summary. Neuro-Oncology. (2021) 23:1231–51. doi: 10.1093/neuonc/noab106. PMID: 34185076 PMC8328013

[2] Buckner JC Shaw EG Pugh SL Chakravarti A Gilbert MR Barger GR . Radiation plus procarbazine, CCNU, and vincristine in low-grade glioma. N Engl J Med. (2016) 374:1344–55. doi: 10.1056/nejmoa1500925. PMID: 27050206 PMC5170873

[3] Mellinghoff IK van den Bent MJ Blumenthal DT Touat M Peters KB Clarke J . Vorasidenib in IDH1- or IDH2-mutant low-grade glioma. N Engl J Med. (2023) 389:589–601. doi: 10.1056/nejmoa2304194. PMID: 37272516 PMC11445763

[4] Goliot N Mohssine S Stefan D Leclerc A Emery E Riverain J . Proton therapy for adult-type diffuse glioma: a systematic review. Crit Rev Oncology/Hematology. (2024) 204:104501. doi: 10.1016/j.critrevonc.2024.104501. PMID: 39251047

[5] Baumert BG Hegi ME van den Bent MJ von Deimling A Gorlia T Hoang-Xuan K . Temozolomide chemotherapy versus radiotherapy in high-risk low-grade glioma (EORTC 22033-26033): a randomised, open-label, phase 3 intergroup study. Lancet Oncol. (2016) 17:1521–32. doi: 10.1016/s1470-2045(16)30313-8. PMID: 27686946 PMC5124485

[6] Bent MJVD Erridge SC Vogelbaum MA Nowak AK Sanson M Brandes AA . Final clinical and molecular analysis of the EORTC randomized phase III intergroup CATNON trial on concurrent and adjuvant temozolomide in anaplastic glioma without 1p/19q codeletion: NCT00626990. J Clin Oncol. (2025) 43:2002. doi: 10.1200/jco.2025.43.16_suppl.2002. PMID: 41909186

[7] Carstam L Latini F Solheim O Bartek J Pedersen LK Zetterling M . Long-term follow up of patients with WHO grade 2 oligodendroglioma. J Neuro-Oncol. (2023) 164:65–74. doi: 10.1007/s11060-023-04368-6. PMID: 37603235 PMC10462563

[8] Weller M van den Bent M Preusser M Le Rhun E Tonn JC Minniti G . EANO guidelines on the diagnosis and treatment of diffuse gliomas of adulthood. Nat Rev Clin Oncol. (2021) 18:170–86. doi: 10.1038/s41571-020-00447-z. PMID: 33293629 PMC7904519

[9] Halasz LM Attia A Bradfield L Brat DJ Kirkpatrick JP Laack NN . Radiation therapy for IDH-mutant grade 2 and grade 3 diffuse glioma: an ASTRO clinical practice guideline. Pract Radiat Oncol. (2022) 12:370–86. doi: 10.1016/j.prro.2022.05.004. PMID: 35902341

[10] Chambrelant I Eber J Antoni D Burckel H Noël G Auvergne R . Proton therapy and gliomas: a systematic review. Radiation. (2021) 1:218–33. doi: 10.3390/radiation1030019. PMID: 30654563

[11] Robbins M Greene-Schloesser D Peiffer AM Shaw E Chan MD Wheeler KT . Radiation-induced brain injury: a review. Front Oncol. (2012) 2:73. doi: 10.3389/fonc.2012.00073. PMID: 22833841 PMC3400082

[12] Winter SF Vaios EJ Shih HA Grassberger C Parsons MW Gardner MM . Mitigating radiotoxicity in the central nervous system: role of proton therapy. Curr Treat Options Oncol. (2023) 24:1524–49. doi: 10.1007/s11864-023-01131-x. PMID: 37728819 PMC12180537

[13] Weber DC Lim PS Tran S Walser M Bolsi A Kliebsch U . Proton therapy for brain tumours in the area of evidence-based medicine. Br J Radiol. (2020) 93:20190237. doi: 10.1259/bjr.20190237. PMID: 31067074 PMC7066950

[14] Gupta A Eisenhauer EA Booth CM . The time toxicity of cancer treatment. J Clin Oncol. (2022) 40:1611–5. doi: 10.1200/jco.21.02810. PMID: 35235366

[15] Goliot N Jouglar E Jacob J Christy F Seutin E Schiappa R . Proton therapy for adult type IDH-mutated glioma: Proglio-1, a multicenter retrospective study. Radiat Oncol. (2025) 20:124. doi: 10.1186/s13014-025-02702-y. PMID: 40764578 PMC12326604

[16] Nagtegaal S van der Weide H Bruynzeel A Lambrecht M Zindler J Rozema T . RADT-45. The multicenter SOPRANO study: outcomes of patients with IDH-mutated WHO grade 2 and 3 glioma treated with proton or photon radiotherapy. Neuro-Oncology. (2025) 27:v102. doi: 10.1093/neuonc/noaf201.0414

[17] Vestergaard A Kallehauge JF Muhic A Carlsen JF Dahlrot RH Lukacova S . Mixed effect model confirms increased risk of image changes with increasing linear energy transfer in proton therapy of gliomas. Radiotherapy Oncol. (2025) 204:110716. doi: 10.1016/j.radonc.2025.110716. PMID: 39809419

[18] Heggebø LC Borgen IMH Rylander H Kiserud C NorDenmark TH Hellebust TP . Investigating survival, quality of life and cognition in PROton versus photon therapy for IDH-mutated diffuse grade 2 and 3 GLIOmas (PRO-GLIO): a randomised controlled trial in Norway and Sweden. BMJ Open. (2023) 13:e070071. doi: 10.1136/bmjopen-2022-070071 PMC10030923 36940951

[19] Slevin F Hudson EM Boele FW Powell JR Noutch S Borland M . APPROACH: Analysis of proton versus photon radiotherapy in oligodendroglioma and assessment of cognitive health - study protocol paper for a phase III multicentre, open-label randomised controlled trial. BMJ Open. (2025) 15:e097810. doi: 10.1136/bmjopen-2024-097810. PMID: 40010843 PMC11865786

[20] Lambrecht M Eekers DBP Alapetite C Burnet NG Calugaru V Coremans IEM . Radiation dose constraints for organs at risk in neuro-oncology; the European Particle Therapy Network consensus. Radiotherapy Oncol. (2018) 128:26–36. doi: 10.1016/j.radonc.2018.05.001. PMID: 29779919

[21] Laperriere N Zuraw L Cairncross G . Radiotherapy for newly diagnosed Malignant glioma in adults: a systematic review. Radiotherapy Oncol. (2002) 64:259–73. doi: 10.1016/s0167-8140(02)00078-6. PMID: 12242114

[22] Adeberg S Harrabi SB Bougatf N Verma V Windisch P Bernhardt D . Dosimetric comparison of proton radiation therapy, volumetric modulated arc therapy, and three-dimensional conformal radiotherapy based on intracranial tumor location. Cancers (Basel). (2018) 10:401. doi: 10.3390/cancers10110401. PMID: 30373115 PMC6266019

[23] Sahm F Capper D Jeibmann A Habel A Paulus W Troost D . Addressing diffuse glioma as a systemic brain disease with single-cell analysis. Arch Neurol. (2012) 69:523–6. doi: 10.1001/archneurol.2011.2910 22158715

[24] Paganetti H Simone CB Bosch WR Haas-Kogan D Kirsch DG Li H . NRG Oncology White Paper on the relative biological effectiveness in proton therapy. Int J Radiat Oncol Biol Phys. (2025) 121:202–17. doi: 10.1016/j.ijrobp.2024.07.2152. PMID: 39059509 PMC11646189

[25] Harrabi SB von Nettelbladt B Gudden C Adeberg S Seidensaal K Bauer J . Radiation induced contrast enhancement after proton beam therapy in patients with low grade glioma – How safe are protons? Radiotherapy Oncol. (2022) 167:211–8. doi: 10.1016/j.radonc.2021.12.035. PMID: 34973277

[26] Werlenius K Agrup M Flejmer A Gojon H Jakobsson F Bergström P . OS05.7.A Radiation-induced contrast enhancement after proton therapy in patients with gliomas in the PRO-CNS study. Neuro-Oncology. (2024) 26:v19. doi: 10.1093/neuonc/noae144.055

[27] Eichkorn T Lischalk JW Horner-Rieber J Deng M Meixner E Kramer A . Analysis of safety and efficacy of proton radiotherapy for IDH-mutated glioma WHO grade 2 and 3. J Neuro-Oncol. (2023) 162:489–501. doi: 10.1007/s11060-022-04217-y. PMID: 36598613 PMC10227167

[28] Acharya S Robinson CG Michalski JM Mullen D DeWees TA Campian JL . Association of 1p/19q codeletion and radiation necrosis in adult cranial gliomas after proton or photon therapy. Int J Radiat Oncol Biol Phys. (2018) 101:334–43. doi: 10.1016/j.ijrobp.2018.01.099. PMID: 29534896

[29] Eichkorn T Lischalk JW Sandrini E Meixner E Regnery S Held T . Iatrogenic influence on prognosis of radiation-induced contrast enhancements in patients with glioma WHO 1–3 following photon and proton radiotherapy. Radiotherapy Oncol. (2022) 175:133–43. doi: 10.1016/j.radonc.2022.08.025. PMID: 36041565

[30] Jaspers JPM Taal W van Norden Y Zindler JD Swaak AT Habraken SJM . Early and late contrast enhancing lesions after photon radiotherapy for IDH mutated grade 2 diffuse glioma. Radiotherapy Oncol. (2023) 184:109674. doi: 10.1016/j.radonc.2023.109674. PMID: 37084885

[31] Bronk JK Guha-Thakurta N Allen PK Mahajan A Grosshans DR McGovern SL . Analysis of pseudoprogression after proton or photon therapy of 99 patients with low grade and anaplastic glioma. Clin Trans Radiat Oncol. (2018) 9:30–4. doi: 10.1016/j.ctro.2018.01.002. PMID: 29594248 PMC5862685

[32] Dworkin M Mehan W Niemierko A Kamran SC Lamba N Dietrich J . Increase of pseudoprogression and other treatment related effects in low-grade glioma patients treated with proton radiation and temozolomide. J Neuro-Oncol. (2019) 142:69–77. doi: 10.1007/s11060-018-03063-1. PMID: 30488294

[33] McMahon SJ Paganetti H Prise KM . LET-weighted doses effectively reduce biological variability in proton radiotherapy planning. Phys Med Biol. (2018) 63:225009. doi: 10.1088/1361-6560/aae8a5. PMID: 30412471

[34] Bahn E Bauer J Harrabi S Herfarth K Debus J Alber M . Late contrast enhancing brain lesions in proton-treated patients with low-grade glioma: clinical evidence for increased periventricular sensitivity and variable RBE. Int J Radiat Oncol Biol Phys. (2020) 107:571–8. doi: 10.1016/j.ijrobp.2020.03.013. PMID: 32234554

[35] El Mjabber R Alami R Filali N Bourial A Dahbi Z Kouhen F . Radiotherapy in Li-Fraumeni syndrome: from biological concern to personalized clinical decision-making. Cureus. (2026) 18:e100978. doi: 10.7759/cureus.100978. PMID: 41658642 PMC12880795

[36] Mayo C Martel MK Marks LB Flickinger J Nam J Kirkpatrick J . Radiation dose–volume effects of optic nerves and chiasm. Int J Radiat Oncol Biol Phys. (2010) 76:S28–35. doi: 10.1016/j.ijrobp.2009.07.1753. PMID: 20171514

