# Supplementary material for: Case Report: Proton beam therapy – friend or foe for patients with IDH-mutated WHO grade 2 and 3 gliomas?
Source: Front Oncol. 2026 May 8;16:1803943. doi: 10.3389/fonc.2026.1803943 (PMC13193975; doi:10.3389/fonc.2026.1803943)
Supplement: Supplementary file 1 [file DataSheet1.pdf]

CARE Checklist of information to include when writing a case report

CARE case report guidelines

(cc) BY-NC-ND

Topic	Item	Checklist item description	Reported on Line
Title	1	The diagnosis or intervention of primary focus followed by the words "case report"	1-2
Key Words	2	2 to 5 key words that identify diagnoses or interventions in this case report, including "case report"	23-24
Abstract (no references)	3a	Introduction: What is unique about this case and what does it add to the scientific literature?	26-31
	3b	Main symptoms and/or important clinical findings	32-39
	3c	The main diagnoses, therapeutic interventions, and outcomes	32-39
	3d	Conclusion—What is the main "take-away" lesson(s) from this case?	40-44
Introduction	4	One or two paragraphs summarizing why this case is unique (**may include references**)	45-77
Patient Information	5a	De-identified patient specific information.	79
	5b	Primary concerns and symptoms of the patient.	79-84
	5c	Medical, family, and psycho-social history including relevant genetic information	79
	5d	Relevant past interventions with outcomes	81-82, 90-94
Clinical Findings	6	Describe significant physical examination (PE) and important clinical findings.	82-84
Timeline	7	Historical and current information from this episode of care organized as a timeline	Figure 2
Diagnostic Assessment	8a	Diagnostic testing (such as PE, laboratory testing, imaging, surveys).	80-84, 95-99, 104-109, 113-120
	8b	Diagnostic challenges (such as access to testing, financial, or cultural)	108-109
	8c	Diagnosis (including other diagnoses considered)	98-104, 116-120
	8d	Prognosis (such as staging in oncology) where applicable	51+82-83
Therapeutic Intervention	9a	Types of therapeutic intervention (such as pharmacologic, surgical, preventive, self-care)	81, 90-93, 99-104, 111-112
	9b	Administration of therapeutic intervention (such as dosage, strength, duration)	90-93, 99-104
	9c	Changes in therapeutic intervention (with rationale)	100-103-104
Follow-up and Outcomes	10a	Clinician and patient-assessed outcomes (if available)	83-84, 87-90, 95-96, 105, 113-114, 116-120
	10b	Important follow-up diagnostic and other test results	106-108, 112-113
	10c	Intervention adherence and tolerability (How was this assessed?)	91, 93-94, 100- 102
	10d	Adverse and unanticipated events	95, 101-102, 105-113
Discussion	11a	A scientific discussion of the strengths AND limitations associated with this case report	165-177
	11b	Discussion of the relevant medical literature **with references**.	136-160
	11c	The scientific rationale for any conclusions (including assessment of possible causes)	161-185
	11d	The primary "take-away" lessons of this case report (without references) in a one paragraph conclusion	186-193
Patient Perspective	12	The patient should share their perspective in one to two paragraphs on the treatment(s) they received	Not applicable
Informed Consent	13	Did the patient give informed consent? Please provide if requested	Yes ☒ No ☐
